# Efficacy and safety of oral SOCG in treatment of major depressive disorder: A protocol for a phase II, randomized, double-blind, placebo controlled, parallel-groups, dose finding exploratory study

**DOI:** 10.1097/MD.0000000000016854

**Published:** 2019-08-30

**Authors:** Ju Yeon Kim, Young Kyung Seo, Ji-Yoon Lee, Weechang Kang, Ik-Seung Chee, Kwang-Yeon Choi, In Chul Jung

**Affiliations:** a Department of Oriental Neuropsychiatry, College of Korean Medicine, Daejeon University; b Department of Data Science, H-LAC, Daejeon University; c Department of Psychiatry, School of Medicine, Chungnam National University, Daejeon, Republic of Korea.

**Keywords:** herbal medicine, major depressive disorder, phase II study, randomized clinical trial, SOCG

## Abstract

**Introduction:**

Major depressive disorder (MDD) is a common condition worldwide, and leads to degradation in quality of life and large socioeconomic costs. There has been increasing demand for new therapies with fewer side effects. SOCG (SOCG tablet) is a modified prescription of So-ochim-tang, which is widely used in Traditional Korean Medicine to treat MDD. We aim to evaluate the efficacy of SOCG in treating MDD, and identify the optimum dose.

**Design:**

The protocol we are following is that of a Phase II clinical trial with a randomized, double blinded, placebo controlled, and parallel design. One hundred forty-eight participants will be randomly divided into 4 groups and treated for 8 weeks.

**Outcome measures:**

The primary outcome will be the score in the Korean Version of the Hamilton Depression Rating Scale. Scores in the Korean version of the Beck Depression Inventory-II Korean Symptom Check List-95 (KSCL-95), State Trait Anxiety Inventory-Korean version, State- Trait Anger Expression Inventory- Korean version (STAXI-K), Insomnia Severity Index (ISI), and the EuroQol-5 Dimension (EQ-5D) will be considered as secondary outcomes.

**Discussion and Conclusions:**

Demonstration of human safety and efficacy of SOCG in the present trial and identification of the appropriate dose will justify a New Drug Application and a phase III clinical trial. Further, we expect that this new antidepressant will be able to increase cure rates, and alleviate the burden of medical expenses.

**Trial registration number:**

Clinical Research Information Service, Republic of Korea (KCT0002763).

## 1 Introduction

Depressive disorders influence general social functions and quality of life, manifesting as depression, lack of concentration, impaired judgement, fatigue, and changes in mood, cognition, and movements.^[1]^ This group of disorders includes disruptive mood disorder, dysregulation disorder, major depressive disorder (MDD), persistent depressive disorder, and various diseases with depression. In general, MDD^[2]^ is considered to be representative of depressive disorders. MDD is defined as single or recurrent episodes of major depression, without episodes of mania or hypomania.^[3]^

The WHO announced that 322 million people have depressive disorders in the year 2015, comprising 4.4% of the world's population.^[4]^ Depressive disorders result in significant social losses including treatment costs, family burden, and workplace inefficiency.^[5]^ Severe obstacles in carrying out social or vocational functions are present, especially if MDD begins in the early 20s to 30s. In Korea, the associated socioeconomic costs have gradually increased to 9.135 billion US dollars in 2011.^[6]^ In the United States, the economic burden of depression was estimated at 187.3 billion US dollars in 2010.^[7]^ MDD is known to relapse in 60% to 70% patients with more frequent episodes being associated with a higher probability of relapse. The recurrence rate 2 years after initial treatment is 25% to 40%, increasing to 60% in 5 years, 75% in 10 years, and 87% in 15 years.^[8]^

However, MDD may have favorable prognosis when properly treated at the early stage. Antidepressants considerably reduce risks of MDD relapse, and comprise a large portion of the pharmaceutical market worldwide, occupying the 3rd place among single drugs and valued at almost 132 million dollars in 2014 in Korea. Further, the demand for anti-depressants is increasing as MDD frequently co-occurs with neuropsychiatric diseases including panic disorder, social phobia, post-traumatic stress disorder, or general anxiety disorder.^[9]^ The market size is expected to gradually expand by 10% to 15% annually.^[10]^ At present, serotonin norepinephrine reuptake inhibitors (SNRIs), noradrenaline reuptake inhibitors (NARIs), and norepinephrine and dopamine reuptake inhibitor (NDRIs) have been developed as antidepressants. In Korea, selective serotonin reuptake inhibitors (SSRIs) are the antidepressants most commonly prescribed by clinicians.^[8]^

SSRIs and tricyclic antidepressants (TCAs) have been reported to have significant antidepressant effects along with various side effects. The most common side effects of SSRIs are nausea, vomiting, diarrhea, stomach ache, hot flashes in the chest, and headache. 10% to 20% of patients experience insomnia, anxiety, and excitement, and 30% to 40% experience sexual dysfunction such as retarded ejection, erectile dysfunction, and decreased sexual desire. TCAs usually cause depersonalization, confusion, orthostatic hypotension, and sedation as initial symptoms and cloudy vision, severe thirst, weight gain, epilepsy, and extrapyramidal symptoms as later symptoms.^[8]^ In addition, Koreans are known to be 4 times poorer metabolizers via the hepatic enzymes compared to western populations, increasing the likelihood of toxicities.^[11]^ Therefore, new antidepressants suitable for use by Koreans are needed. In this regard, natural products have recently been receiving attention which have been due to their proven safety and effectiveness based on clinical experience. Herbal decoctions described in various Korean classical medical texts have shown effectiveness in treating MDD in clinical settings.

SOCG is a prescription containing So-ochim-tang combined with *Platycodonis Radix* and *Aurantii Fructus Immaturus*. So-ochim-tang is widely used for treatment of MDD and somatization disorder, which are thought to be caused due to stagnation of qi. Its use is mentioned in “Donguibogam”^[12]^ (“So-ochim-tang for … every qi-related problem”) to treat qi-related pains in the chest and abdomen. SOCG has been shown to have reasonable antidepressant activity in animal behavior studies compared to different antidepressant treatments. It has also been patented as a composition for treating depressive disorder^[13,14]^ (Registered patent of Republic of Korea 10-1910013). However, clinical effects in humans through well-designed trials have not been assessed.

In the present study, we aim to assess the efficacy and safety of orally administered SOCG in MDD patients and determine the appropriate dosage.

## 2 Methods and materials

### 2.1 Study design and setting

The study is designed as a Phase II clinical trial and is following a randomized, double blinded, placebo controlled, parallel design to examine the efficacy of SOCG in MDD treatment.

Participants will be selected from volunteers based on demographic information, medical history, vital signs, lab examinations, and selection criteria. Selected participants will be assigned to 3 experimental groups and 1 placebo group according to the stratified block randomization. The experimental groups will be a low dose group, a medium dose group, and a high dose group. Participants will be given SOCG or placebo 3 times/day, for 8weeks (56 days). They will be examined using the Korean Version of the Hamilton Depression Rating Scale (K-HDRS), the Korean version of the Beck Depression Inventory-II (K-BDI-II), Korean Symptom Check List-95 (KSCL-95), State Trait Anxiety Inventory-Korean version (STAI-K), State Trait Anger Expression Inventory Korean version (STAXI-K), Insomnia Severity Index (ISI), Instrument on Pattern Identifications for Depression, EuroQol-5 Dimension (EQ-5D), and melatonin and cortisol levels to assess score variation, improvements in quality of life, and biological indicators.

**Figure 1:**
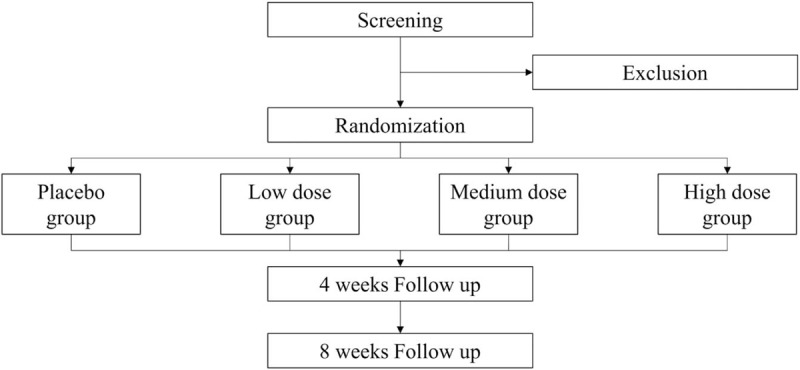
The study design flowchart.

The research progress is summarized in Figure 1 and the examination details followed by visit schedules are shown in Table 1. Each visit should occur at the scheduled date or within ±4 days. The screening tests will be completed 10 days before the second visit (0 week). The results will be examined at the second visit. The lab tests will include hematologic tests for levels of hemoglobin, hematocrit, red blood cell count, white blood cell count, platelet count, and erythrocyte sedimentation rate, and hematochemical tests for levels of aspartate aminotransferase (AST), alanine aminotransferase (ALT), gamma-glutamic transpeptidase (γ-GTP), alkaline phosphatase (ALP), blood urea nitrogen (BUN), creatinine, total bilirubin, glucose, Na+, K+, Cl-, lactate dehydrogenase (LDH), creatine phosphokinase (CPK), and urine tests including dip-stick test and urine microscopy. Urinary human chorionic gonadotropin (hCG) level will be checked 14 days before the second visit for every participant with the possibility of pregnancy, experiencing lack of menstruation for less than 1 year since the last menstrual period.

**Table 1:**
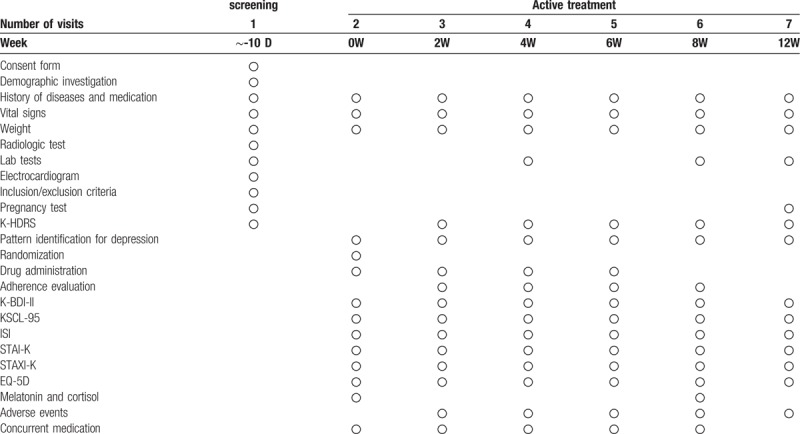
The schedule of SOCG clinical trial.

### 2.2 Participants and recruitment

One hundred forty eight participants in total will be competitively recruited in the Dunsan Korean medicine hospital of Daejeon university and the Chungnam national university hospital. Recruitment will begin in December, 2018 via advertisements in Daejeon subway stations, Dunsan Korean medicine hospital of Daejeon university, and Chungnam national university hospital.

#### 2.2.1 Eligibility criteria

Inclusion criteria

(1) Age between 19 and 65 years.
(2) Diagnosis of MDD per the Diagnostic and Statistical Manual of Mental Disorders – 5th edition (DSM-5).
(3) Score >22 per the K-HDRS.
(4) Decision to participate in the study and voluntary consent by signing the consent form.

Exclusion criteria

(1) Suicide risk.
(2) Requirement of hospitalization.
(3) Delusions, hallucinations, psychotic symptoms, or such a history.
(4) Manic episodes, hypomanic episodes, mixed episodes or such a history.
(5) Alcohol or other substance abuse/dependency issues or such a history.
(6) Use of substances or drugs that may affect MDD symptoms (antianxiety drugs, antidepressants, antipsychotics, adrenocorticotrophic hormone drugs, estrogen preparations, L-dopa, digitalis, bromide, cyclosporine, disulfiram, isoniazid, yohimbine etc), unless 4 weeks have passed since the last use.
(7) Hypokalemia or hypokalemia-inducible diseases (hypomagnesemia, Bartter syndrome, Gitelman syndrome, hyperaldosteronism inducible diseases, etc) or use of hypokalemia-inducible drugs (furosemide, thiazide, amphotericin, cisplatin, etc).
(8) Use of warfarin.
(9) Diagnosis of cardiac arrhythmia
(10) Medical conditions that may affect the MDD symptoms (myocardial infarction, brain tumor, multiple sclerosis, pancreatic disease, hypo/hyperthyroidism, hyperparathyroidism, Addison's disease, Cushing's disease, Rheumatoid arthritis, cancer, cerebrovascular disease, dementia, epilepsy, etc).
(11) Chronic diseases resistant to treatment (chronic active hepatitis, hypertension, diabetes mellitus, etc).
(12) Treatment for hepatic tumor, hepatic cirrhosis, chronic renal failure, or congestive heart failure classified as New York Heart Association Class (NYHA Class) III–IV
(13) Disabilities of hepatic or renal function (ALT, AST, ALP ≥ twice the upper limit of normal or creatinine > 2.0 mg/dL at screening).
(14) Galactose intolerance, Lapp lactase deficiency, glucose-galactose malabsorption, or genetic problems.
(15) Diseases that may influence drug absorption or digestive problems after associated surgery.
(16) Participation in other clinical trials within the prior month.
(17) History of hypersensitive or allergic reactions to drugs involved in the study.
(18) Use of other herbal decoctions or extracts.
(19) Difficulties in understanding the process of consent or in continuing to participate in the study due to mental retardation or cognitive problems.
(20) Pregnancy or lactation.
(21) Possibility of pregnancy and refusal to use contraceptive methods including double contraception^∗^, intrauterine contraceptive device, or spermicides during the study. ∗Double contraception is the use of barrier contraception (condom, diaphragm, etc) with other methods (sterilization surgery, intrauterine contraceptive device, contraceptive cream, jelly or foam).
(22) Unsuitability for participation in the study due to other reasons.

### 2.3 Randomization and blinding

One hundred forty eight participants will be equally divided into 4 groups. We will use stratified blocked randomization with the clinical trial institution as stratification variable. Each group will be distinguished using specific signs used in the randomization table. The signs and block size will not be disclosed. The randomization table will be re-creatable.

The product manufacturer Han kook shin yak corporate will prepare tablets based on the randomization tables. The tablets will be labeled as “Institution code-R-serial number” to be offered to the chief investigator and managed by the pharmacist responsible. The table matching the randomization table in terms of the serial numbers will be sealed and managed by the chief investigator. The seals will be amenable to examination to ascertain whether they have been removed. When the study physician requests administration of the preparation, the pharmacist will check the institution code and the serial number to assign the medication. The identification code will be recoded on the serial number table and managed by the pharmacist.

The participants, chief investigator, study physician, and coordinator will be unable to recognize the type of medication. Elimination of the blinding will only be considered in a severe medical emergency. When the investigator notices an absolute need for an emergency situation, the chief investigator will decide whether to eliminate the blinding through discussion. The chief investigator will be expected notify the removal and explain the detailed emergency situation to Institutional Review Boards (IRBs) of each institution within 24 hours.

### 2.4 Interventions

SOCG is a brown rectangular film-coated tablet, containing 395 mg of SOCG soft extracts (Cyperi Rhizoma, Linderae Radix, Aucklandiae Radix, Glycyrrhizae Radix et Rhizoma, Platycodonis Radix, and Ponciri Fructus Immaturus). The control drug is an identically shaped tablet, containing only excipients.

SOCG or placebo will be administered orally, with water, 30 minutes after each meal, 3 times a day for 8 weeks (56 days).

Treatment groups:

(1) Low dose group: 1 tablet of SOCG and 2 tablets of placebo.
(2) Medium dose group: 2 tablets of SOCG and 1 tablet of placebo.
(3) High dose group: 3 tablets of SOCG.

Control group: placebo

(1) Placebo group: 3 tablets of placebo.

### 2.5 Outcome measures

#### 2.5.1 Primary outcome

The primary outcome will be changes in the K-HRDS score, comparing the baseline score and the last observed score during the eight weeks of treatment.

#### 2.5.2 Secondary outcomes

① Recovery of the K-HDRS (The percentage of participants whose K-HDRS improved >50%): Recovery in the last observed K-HDRS during the 8 weeks of treatment.

② Remission of K-HDRS (The percentage of participants whose K-HDRS improved to a score <7): remission in the last observed K-HDRS during the 8 weeks of treatment.

③ K-BDI-II: Changes in K-BDI-II comparing the baseline and the last observed score during the 8 weeks of treatment.

④ KSCL-95: Changes in KSCL-95 comparing the baseline and the last observed score during the 8 weeks of treatment.

⑤ ISI: Changes in ISI comparing the baseline and the last observed score during the 8 weeks of treatment.

⑥ STAI-K: Changes in STAI-K comparing the baseline and the last observed score during the 8 weeks of treatment.

⑦ STAXI-K: Changes in STAXI-K comparing the baseline and the last observed score during the 8 weeks of treatment.

⑧ Instrument on Pattern Identifications for Depression: Changes in Instrument on Pattern Identifications for Depression score, comparing the baseline and the last observed score during the 8 weeks treatment.

⑨ EuroQol-5 dimension (EQ-5D): Changes in the EQ-5D comparing the baseline and the last observed score during the 8 weeks of treatment.

10. Melatonin & Cortisol Study: Changes in the concentration of melatonin and cortisol comparing the baseline and the last observed score during the 8 weeks of treatment.

#### 2.5.3 Safety evaluation

Every participant given the medication at least once will be assessed for occurrence of adverse events and their association with the medication. Potential adverse events include all medical symptoms that will occur during the trial. Every unintended phenomenon, side effects, abnormalities in vital signs, and lab tests will be documented in an adverse events report. The investigator responsible will assess the adverse events at different stages based on their seriousness. The adverse events will be documented to be communicated to the participants and/or their legal representatives immediately and will be notified to the IRBs.

### 2.6 Analysis of adherence

Use of the medications will be recorded at each visit for assessing drug compliance and documented in the case report form. Drug compliance will be estimated as follows. 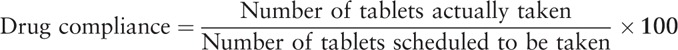


### 2.7 Statistical methods

#### 2.7.1 Sample size

The sample size required is based on results of the t-test for independent samples comparing changes in the K-HRDS score from baseline to the last observed one during the 8 weeks of treatment between the SOCG medium dose group and the placebo group.

Because no relevant results of clinical trials have been reported, the sample size was calculated referring to a clinical trial of similar design involving patients with depression.^[15]^

The sample size of each group is 37, considering a significance level of 5%, a power of 80%, and assigning a ratio of 1:1:1:1 and a dropout rate of 5%.

#### 2.7.2 Statistical analysis

The efficacy will be evaluated based on intention-to-treat (ITT) analysis and per-protocol (PP) analysis. Participants with total drug compliance <75% will be excluded from PP analysis.

Continuous data will be represented by mean, standard deviation, minimum, and maximum and categorical data will be represented by a frequency table. We will compare data using analysis of variance (ANOVA) for the continuous variables, and Pearson chi-squared test or Fisher's exact test for the categorical variables. The size of the type I error caused due to multiple comparisons will be modified using Bonferroni's method.

The primary outcome, the changes in the K-HRDS, will be evaluated using analysis of covariance (ANCOVA) with the experimental group, baseline K-HDRS, and the institution as the explanatory variables. The estimates of mean differences between placebo and each SOCG group, the 95% confidence interval, and the *P*-value will be presented. For evaluating the sensitivity of missing values of K-HDRS, the primary outcome observed at each point will be analyzed using the linear mixed model. The explanatory variables include all groups, baseline K-HDRS, institutions, the observation time point, and the interaction term of the experimental group based on the observation time point.

Among the secondary outcomes, recovery rate and remission rate of K-HDRS will be analyzed using logistic regression analysis with the experimental group, baseline K-HDRS, and the institution as the explanatory variables. Estimates of odds ratio of each SOCG group, 95% confidence interval, and *P*-value will be presented using the placebo group as the standard category. The other secondary outcomes will be analyzed using ANCOVA with the experimental group, baseline outcomes, and the institution as the explanatory variables.

For safety assessment, outcomes of lab tests will be compared to the baseline values. Each adverse event will be listed with a detailed explanation, and the frequency will be recorded separately based on correlation with the medication. Adverse events will be summarized based on groups and organs. The ANOVA or Kruskal-Wallis test will be used to compare the frequency of medication-correlated adverse events between groups. The Pearson chi-squared test or Fisher's exact test will be used to compare the percentage of participants who experienced the adverse events at least once between the groups.

### 2.8 Data management and monitoring

All documents and records will be preserved in highly secured document storage rooms in the institutions for 3 years from the end of the trial. Monitoring will involve regular checkups to investigate if the clinical trial is following the protocols, standard procedures, administrative standards, and regulations, and the management of the entire process. The monitoring will also include regular visits and calls. The staff will check the original records of the participants and the preservation of the documents. The staff will also closely review the trial process and discuss every problem with the investigators.

### 2.9 Ethical considerations, informed consent, and study registration

This clinical trial protocol was approved by the IRB of Dunsan Korean medicine hospital of Daejeon university and Chungnam national university hospital (Approval number; DJDSKH-17-DR-35, CNUH 201803050001-HE006) and will be performed following all applicable regulations.^[16–21]^ The chief investigator will notify the IRBs when the protocol needs any modification. Informed consent of the patients will be obtained prior to conducting any process in the clinical trial.

All records of participants will be kept confidential until publication. The clients and monitoring staff can request the records only for monitoring or management. Signing the consent form indicates that the participants are consenting to providing access to clients and staff to review and copy their documents in terms of both legal or ethical aspects. This information will be kept confidential. All documents will be recorded and distinguished based on the participant's code.

## 3 Discussion

MDD is a common psychiatric disorder with increasing prevalence worldwide.^[22]^ Several antidepressants have been marketed and used but are associated with many side effects, raising the need for safe therapies with fewer side effects.

In Korean traditional medicine clinical practice, the Ul-syndrome (Yuzheng) category corresponds to depression.^[23]^ It is caused due to mental stress and its clinical manifestations include stagnation of qi, insomnia, anorexia, irritability, nervousness, grief, and stuffiness. Various herbal medicine prescriptions have been used and have shown positive effects.

We conducted animal behavior studies using various herbal prescriptions used to treat Ul-syndrome, and found that So-ochim-tang-gamibang (SOCG) had a significant antidepressant effect.

So-o-chim-tang has been used to treat depressive mood and somatic pain by relieving qi stagnation for the last two decades. SOCG is modified from So-ochim-tang by adding *Platycodi Radix* and *Aurantii Fructus* based on Korean traditional medical theory. These additives are thought to increase efficacy by helping qi circulation.^[24,25]^ Based on the premise that a prolonged stagnation of qi and blood flow, and an imbalance in yin–yang causes the symptom of depression, SOCG or modified decoctions of SOCG have been prescribed in traditional medicinal therapy.^[26]^

We planned the present trial to determine the efficacy and safety of SOCG in treating patients with MDD. K-HDRS will be used as the inclusion criterion and outcome measure. K-HDRS is one of the most commonly used measures of depression.^[27–29]^ We will use the K-HDRS as translated by Yi et al^[30]^ in 2005. The 17 items of K-HDRS are depressed mood, feelings of guilt, suicide, early insomnia, middle insomnia, late insomnia, work and activities, retardation, agitation, psychic anxiety, somatic anxiety, gastrointestinal somatic symptoms, general somatic symptoms, genital symptoms, hypochondriasis, and loss of weight and insight. The score is from 0 to 52 points, and the higher the score, the more severe the depression.^[31]^

We will include those with a K-HDRS of 22 or higher in the inclusion criteria. This is intended for those with moderate to severe severity, because the study is a Phase II clinical trial with an aim to clearly confirm efficacy in MDD treatment and to determine dosage.

Changes of the degree of depression will be assessed based on score changes before and after the study. In addition, the percentage of subjects showing an improvement of more than 50% in the K-HDRS score (recovery rate) and the percentage of patients improving to a score less than 7 points (remission rate) will be assessed. Further, at 4 weeks after the end of the test, follow-up studies will be conducted to determine the persistence of efficacy.

The Beck Depression Inventory (BDI) is one of the most widely used self-reported scale in the world. It is commonly used as a tool for screening patients in the general population who are likely to develop depression or for selecting subjects for studies as well as diagnosing and evaluating treatment effects.^[31]^ In 1996, BDI-II^[32]^ was developed as a revised criteria of depression per DSM-IV. The Korean version (K-BDI-II) was standardized by Sung et al^[33]^ in 2008, and the reliability and validity were found to be very high. In the present study, we will assess the severity of symptoms using this tool.

Clinicians always consider suicidal risks when treating depression. One of the major symptoms of depression is presence of suicidal thoughts or impulses. Likewise, most cases of suicides are associated with a history of depressive symptoms.^[34,35]^ In the present clinical trial, suicidal ideation and behavior will be identified through interviews and participants with suicidal risk will be excluded per ethical guidelines.

The KSCL-95 was developed by Kwon^[36]^ by modifying SCL-90-R to reflect the current mental health environment and socio-cultural characteristics of Korean society. It is a comprehensive test that can measure major clinical psychological symptoms. We will investigate changes in general mental characteristics of the subjects using this tool.^[37]^

There is a significant correlation between sleep and depressive symptoms.^[38]^ It has been reported that the risk of clinically severe depression is 9.82 times higher in insomnia patients than in the general population.^[39]^ MDD is the most frequent psychiatric disorder associated with insomnia.^[12]^ ISI is a tool to evaluate sleep quality and insomnia. It helps in assessing the severity of insomnia, satisfaction with current sleep patterns, interruption in daytime functioning, manifestation of damage due to sleep disorders, and pain caused due to sleep.

STAI-K is a useful test to identify anxiety in clinically anxious groups and psychiatric patients. It assesses state anxiety and trait anxiety.^[40]^ State anxiety and trait anxiety are often accompanied by depression.^[41]^

STAXI-K is designed to assess anger experience and anger expression style. It consists of ten items assessing state anger and trait anger, and eight items assessing anger control, anger expression, and anger suppression.^[42]^

The EQ-5D is a general assessment tool for health-related quality of life developed by the EuroQol group. It includes the 5 items of mobility, self-care, usual activities, pain/discomfort, and anxiety/depression.^[43,44]^

Traditional Korean Medicine uses a diagnostic tool called Pattern Identification. Pattern Identification is a method of comprehensive analysis of the information collected through the four stages of diagnosis; visual inspection, interrogation, auscultation, palpation, and the various symptoms.^[45]^ One of its advantages is that it can be used as a basis for treatment to clarify the nature of the pathology more clearly.^[46]^

The Pattern Identification Tool has been found to be reliable and valid in assessing depression.^[47]^ It assesses the 6 parameters of stagnation of liver Qi, dual deficiency of the Heart and Spleen, Qi-deficiency mingled with phlegm, relieving stagnation of phlegm-Qi, stagnation Qi transforming into fire, and Yin deficiency with effulgent fire. This is a structured interviewing tool, and each pattern identification score is calculated by substituting the measured value into the calculation formula. Thus, it is possible to confirm the type or tendency of pattern identification.

Melatonin is a hormone that regulates the biological cycle of an individual, and its levels are inhibited during the daytime and gradually increase at night.^[48]^ Studies on the relationship between melatonin levels and psychiatric disorders have been carried out in a variety of ways, and studies have shown that low levels of melatonin at night cause dysthymia and depression.^[49,50]^ Melatonin can be detected in the saliva, blood, and urine.^[51]^ The saliva method is a non-invasive method capable of evaluating the melatonin cycle in real time.^[52]^ Because the concentration of melatonin measured in blood is approximately 3 times that in saliva,^[53]^ methods for measuring blood levels based on saliva concentrations are widely used.

Cortisol is a reliable biomarker that most accurately represents the altered biochemical state in response to stress stimuli.^[54]^ Carroll et al^[55]^ reported that cortisol levels in patients with MDD were two times higher than those in normal subjects. Cortisol also has a circadian rhythm, which is highest in the early morning and lowest in the evening between 8 PM and 2 in adults.^[56]^ Cortisol can also be measured in saliva.

Saliva will be collected using the Salivettes system (Sarstedt, Germany) and the amount of melatonin and cortisol secretion will be measured. Saliva will be collected in a precise manner and stored frozen (−20°C) immediately without contamination. The subject will visit the trial center at a specific time (10 AM ± 1 hour) during visit 1 and visit 6. We will examine the differences in the levels of these 2 hormones in the subjects in comparison to normal values.

Results of preclinical examinations performed in rats thus far have shown the presence of colored stool, dilute stool, mucous stool, diarrhea, and salivation, but no toxicologically significant effects were observed due to SOCG administration. SOCG has not been tested on healthy individuals or patients, and therefore no side effects have been clearly revealed. However, the possibility of some side effects remains, including dyspepsia, diarrhea, dizziness, rash, fatigue, which are usually found to be occurred due to herbal medicines. The chief investigator and the investigator responsible will pay detailed attention to the safety of the participants and minimize any potential damage by carrying out prompt and appropriate measures when severe adverse events occur. In addition to the predicted adverse events, the possibility of emerging new symptoms cannot be ruled out and prompt action will be taken in such cases.

The recruitment of subjects started in November 2018 and will be completed by December 2020. The results will be announced within a few months of trial completion. Based on the study, if human safety, efficacy and the appropriate dose of SOCG are confirmed, this can be used as preliminary data supporting the utility of this Korean traditional medicine in treating depression, and to obtain a New Drug Application (NDA) for a phase III clinical trial. Further, we expect that this new antidepressant medication will be able to raise the depression cure rate among Koreans, alleviate the burden of medical expenses, and help revive the Korean medical herb industry.

## Author contributions

**Conceptualization:** Ik-Seung Chee, Kwang-Yeon Choi, In Chul Jung.

**Methodology:** Weechang Kang.

**Supervision:** In Chul Jung.

**Writing – original draft:** Ju Yeon Kim, Young Kyung Seo.

**Writing – review & editing:** Ju Yeon Kim, Young Kyung Seo, Ji-yoon Lee.
